# Human papilloma virus and cervical preinvasive disease

**Published:** 2009-11-25

**Authors:** G Peltecu, M Bari, G Iancu, F Popa

**Affiliations:** * Filantropia Hospital of Obstetrics and Gynecology and Carol Davila University of Medicine and Pharmacy, BucharestRomania; ** St. Pantelimon Hospital, Department of Surgery, Carol Davila University of Medicine and Pharmacy, BucharestRomania

**Keywords:** HPV, cervical cancer, cervical preinvasive disease, prevention, vaccine, conization

## Abstract

Cervical cancer lesions represent a major threat to the health of the women worldwide. Human Papillomavirus (HPV) is responsible for 99.7% of cervical cancer cases, the infectious etiology giving the possibility of preventing cervical cancer by vaccination. The most aggressive HPV types are 16 and 18, which cause about 70% of cases of invasive cancer. The vaccination is recommended to the girls aged 11–12. The diagnosis and the treatment of cervical preinvasive disease allow the doctor to prevent the development of the invasive disease.

## Introduction

Cervical cancer and precancerous cervical lesions constitute a major health problem for the women. The clinical and molecular studies have identified the HPV as the main cause of cervical preneoplastic lesions and cervical cancer. Almost all cervical cancers contain genetic material from the high–risk HPV types. The screening has dramatically reduce the incidence of the cervical cancer. However, in the developing countries where the access to the medical services is poor, the cervical cancer has a very high incidence, being in the second place as a cancer related cause of death [1]. The treatment of preinvasive disease consists in ablative or excisional procedures, with approximately 90% of effectiveness, with low morbidity and costs, compared to the treatment of invasive disease. The preventive vaccination tries to eradicate the infection by protecting the young girls before the encounter with the virus.

## Pathogenesis

The history of the HPV began in the seventies when zur Hausen isolated the type 6 from the warts lesions. Since then, HPV is recognised as the etiologic agent of the cervical cancer. Until now, there are over 40 viral strains implicated in the pathogenesis of cervical cancer.

HPV belongs to the family of the Papovaviridae. The viral infection is a necessary factor for the appearance of the cervical neoplasia, but it is not enough, requiring also particular host and environmental factors. The HPV infection seems to be implicated in 99.7% of the cervical cancer. Virtually all cervical cancers (over 99%) contain the genes of high–risk HPV, especially 16, 18, 31 and 45. Most of HPV infections regress spontaneously, especially those in adolescents and young women [2].

There are over 200 types of HPV, divided in three categories: the high–risk types (generating HG–SIL lesions and invasive neoplasia)–16, 18, 31, 33, 35, 45, 52, 56, 58, 59, probably high–risk types– 26, 53, 66, 68, 73, 82 and low–risk (moderate dysplasia and genital warts)–6, 11.

The vaccine against the infection with HPV has made possible the cervical cancer primary prophylaxis. The secondary prophylaxis was found also very effective. The cervical dysplasia cure is accurately assessed by the combination of negative PAP smear and negative DNA–HPV test.

The majority of HPV infections are subclinical and transitory (>80% disappear within 2 years) due to the cellular immune response. After infection, HPV is latent for 2–12 months. The cellular immune response starts in about 3 months from inoculation of virus and eliminates or suppresses it down to undetected levels.

In some subjects, the infection can cause warts (condyloma) and low–grade cervical lesions. Sometimes, in certain cases, the high risk HPV types (16, 18, 31, 45) can be persistent and progress to high–grade lesions and cervical cancer in a few years [3]. The mean interval of natural progression to invasive cancer is about 13 years.

HPV infection means the viral particles replicate in the mature keratinocytes. Active infection occurs when the virus invades the basal epithelial layer. Persistency of infection means the HPV infected the stem epithelial cells. Viral replication needs the oncoproteins E6 and E7 which bind the tumor supressor genes p53 and pRB. The initiation of oncogenic process begins when viral DNA was integrated in the host genome [4]. Viral transmission is done particularly through sexual intercourse. Use of condoms lowers the risk of transmission, but doesn't offer complete protection.

Until the age of 50, it is estimated that over 80% of sexually active women became HPV infected at some moment in their lives. Approximately 75% of the new HPV infections appear between the ages 15 and 24. Most of these infections are transient and disappear spontaneously (70% at one year, 90% at 2 years for young women). HIV coinfection, which suppresses the cellular immune response, represents a severity factor. The time from the first HPV infection, persistency, preneoplastic lesions and cancer is estimated to be 13–15 years.

## Diagnosis

Diagnosis of preneoplastic lesions is based on cytology, colposcopy and the final diagnosis is established by histopathological examination of the tissue sample.

### Cytologic examination.*Bethesda* System

The Bethesda System and its 2001 revisions aim to simplify Papanicolaou (Pap) smear reporting and to make it more reproducible. The main types of preneoplastic lesions described are: atypical squamous cells of undetermined significance (ASCUS); atypical squamous cells, cannot exclude high–grade squamous intraepithelial lesion (ASC–H); low–grade squamous intraepithelial lesion (LSIL), high–grade squamous intraepithelial lesion (HSIL), squamous cell carcinoma, atypical glandular cells (AGC), endocervical adenocarcinoma in situ (AIS) and adenocarcinoma [5] (Table 1).

**Table 1:** The 2001 Bethesda System for Reporting Cervical Cytologic Diagnoses

**Specimen type**
Conventional smear (Pap smear)
Liquid–based preparation
**Specimen adequacy**
Unsatisfactory for evaluation
**Interpretation of the results**
A. Negative for intraepithelial lesion or malignancy
1. Microorganisms (Trichomonas, Candida, Gardnerella etc.)
2. Other non–neoplastic findings (inflamation or reactive cellular changes; radiotherapy; IUD; atrophy, glandular cells status post hysterectomy)
B. Other endometrial cells (in woman more than 40 years of age)
C. Epithelial cell abnormalities:
1. *Squamous cell*
Atypical squamous cells (ASC)
–ASC of undetermined significance (ASC–US)
–ASC, cannot exclude high–grade squamous intraepithelial lesion (ASC–H)
Low–grade squamous intraepithelial lesion (LSIL)
– encompassing: HPV, mild dysplasia, and CIN
High–grade squamous intraepithelial lesion (HSIL)
–encompassing: moderate and severe dysplasia, carcinoma in situ, CIN Ⅱ, and CIN Ⅲ
Squamous cell carcinoma
2.*Glandular cell*
Atypical glandular cells (AGC)
–specify endocervical, endometrial, or glandular cells not otherwise specified (NOS)
Atypical glandular cells, favor neoplastic
–specify endocervical or not otherwise specified (NOS)
Endocervical adenocarcinoma in situ (AIS)
Adenocarcinoma
D.Other malignant neoplasms

### Colposcopy

Colposcopy identifies a series of lesions associated with preneoplastic cervical lesions (acetowhite changes, leukoplakia, punctation and mosaic). *Acetowhite changes* represents the white zone that appears after treatement of the cervix with acetic acid (3–5%). The acetic acid does not affect the mature epithelium rich in glycogen, but colours the dysplasic epithelium in white because of the high protein content. The metaplasic epitelium is very thin–unlike CIN which becomes white–so it will become gray and translucent. *Leukoplakia* (white epithelium after acetic acid aplication)–hyperkeratosis–is the most frequent cause of HPV infection. *Punctations* (a zone of red dots–dilated capilaires which ends to the surface) in well circumscribed areas indicates an abnormal epithelium, most frequent CIN. *Mosaicism* (an abnormal pattern of interconnecting small blood vessels) is associated with high grade lesions–CINⅡ/Ⅲ [6].

### Histopathology

In mild dysplasia (CIN Ⅰ) only few cells in the basal third of the epitelium are abnormal, while in moderate dysplasia (CINⅡ) the abnormal cells involve about 2/3 of the thickness of the surface lining of the cervix. In severe dysplasia or carcinoma in–situ (CINⅢ) the entire thickness of the basal epithelium is abnormal. Spontaneous regression rate of CIN Ⅰ is about 60–85%.The regression usually solves in about 2 years. All CINⅡ and CIN Ⅲ lesions need treatment. The progression CINⅡ to CIS is 20% and invasion in about 5% of patients [7].

### Vaccination

The characteristic of the cervical cancer is its infectious etiology and the possibility of preventing the cancer through vaccination against HPV, the central factor implicated in pathogenesis.

Speaking about prevention strategies, we can separate them in three categories:

1. ***Primary prevention*** –prophylactic vaccination and modification of risk factors (behavioural factors and at risk sexual behaviours).
2. ***Secondary prevention*** –early detection and treatment of the preneoplastic lesions (screening programs for CIN and HPV, excisional therapy, therapeutic vaccines, retinoids, immune modulators).
3. ***Tertiary prevention***–cervical cancer treatment and postsurgical follow–up (surgery, chemotherapy, radiotherapy and posttreatment follow–up).

Two vaccines are at the moment licensed in USA and Europe: a bivalent one and a tetravalent one. Both of them are using the same principle: they contain particles identical to the viral capsid, without any viral DNA, lacking the infectivity or the oncological risk. The empty protein shells are composed of major L1 capsid proteins which are specific for each HPV type [8]. The tetravalent vaccine protects against HPV types 6, 11, 16 and 18 and the bivalent vaccines against types 16 and 18. The vaccines are given as three intramuscular shots at 0, 2 and 6 months for the tetravalent vaccine and at 0, 1 and 6 months for the bivalent vaccine. The side effects reported have been minor, consisting of mild local and systemic reactions. Fever has been the most commonly systemic reaction and pain, the most commonly local reaction. Other adverse reactions reported were locally erythema and edema and nausea and headaches [9]. Both vaccines are effective; at over 5 years from the beginning of the vaccination, the effectiveness of both vaccines was close to 100% in preventing persistent infection with HPV type 16 and 18 and CIN Ⅱ and Ⅲ associated to these types. The tetravalent vaccine protects also against genital warts, vaginal and vulvar intraepithelial neoplasia [10]. The vaccination is recommended for the girls ages 11–12 and could begin from the 9 years of age. The vaccination is also recommended for the patients between 13 and 18 years old for the completion of the vaccination scheme. Untill now, proof of efficacy lacks for the vaccination of women aged 19 to 26. Screening for preneoplastic lesions and cervical cancer must continue in the general population, but also in the vaccinated population [11, 12].

### The management of the precursor lesions of the cervical cancer

There are a few categories for whom the management is a little bit different because of their characteristics: adolescents (aged <20), pregnant women, postmenopausal women and immune–compromised women. Adolescents have a higher prevalence of the HPV infection, more frequent minor lesions and a very low risk of cervical invasive cancer compared to older patients, because most of the HPV infections disappear spontaneously in 2 years of time and have little short term significance. Therefore, the colposcopy should not be performed as a first line investigation. During pregnancy, endocervical curettage is forbidden, while the colposcopic guided biopsy is indicated for HSIL or when invasive cancer is suspected. Colposcopy is a reasonable investigation for the low risk pregnant women [13].

### ASC–US

High risk DNA–HPV testing, repeated cervical smear and colposcopy are all acceptable methods in the management of ASC–US lesions for the patients aged over 20 years. The patients ASC–US and DNA–HPV negative for the high risk types will be followed up at 12 months. Patients with ASC–US and DNA–HPV positive should be treated as the patients with LSIL and evaluated by colposcopy. Endocervical curettage is preferred for the patients without colposcopic lesions or with unsatisfactory colposcopy. The management after colposcopy for ASC–US/DNA–HPV positive patients, which do not have CIN diagnosed, consists in repeating the DNA–HPV test every 12 months or repeating Pap smear every 6–12 months. DNA– HPV testing is not recommended earlier than 12 months. Repeating cytology at 6 months is indicated until two consecutive negative results are obtained, then normal follow–up is recommended [8]. Colposcopy is recommended for patients with ASC–US or severe lesions at the repeated testing, without taking into account DNA–HPV status.

Excisional diagnostic procedures are not recommended in the treatment of the patients with ASC–US as an initial diagnosis, in the absence of CIN Ⅱ/Ⅲ histopathological diagnosis.

### ASCH

The management of ASC–H implies the colposcopic evaluation from the beginning. If there is an evident colposcopic lesion, biopsy is recommended. For the patients without CIN Ⅱ/Ⅲ, the follow–up is acceptable by testing DNA–HPV every 12 months or repeating cytology every 6–12 months. If DNA–HPV testing is negative or two consecutive smears are negative, return to normal follow–up is recommended.

### LSIL

Colposcopy is recommended from the beginning for LSIL cytology. Endocervical curettage is preferred for the patients without colposcopic lesions or with unsatisfactory colposcopy. After colposcopy, for the patients with LSIL without histopathological CIN Ⅱ/Ⅲ, the recommended attitude is DNA–HPV testing at every 12 months or repeated cytological at 6–12 months. If the DNA–HPV testing or two successive cytological results are negative, normal follow–up is recommended. If HPV DNA tesing is positive or at least one cytological testing result is ASC–US or higher, colposcopy have to be repeted.

### HSIL

For HSIL lesions the risk for preinvasive disease is 70–75% and for invasive disease is 1–2% [14]. So, colposcopy and biopsy are indicated ab initio. Endocervical evaluation (cytological, colposcopic or curettage) is mandatory, excepting the case when the excisional biopsy is taking into account. When colposcopy and endocervical cytological evaluation are negative, the diagnosis of HSIL is reconsidered. If the cytological result persists, excisional biopsy is recommended [14].

### AGC

Fractioned curettage (endocervical and endometrial) and colposcopy are recommended for the patients with AGC/AIS aged over 40 years old. Also endometrial curettage is to be done for the patients over 40 years old with risk factors for endometrial cancer. DNA–HPV testing is not routinely recommended. The most frequent viral types associated with endocervical adenocarcinoma are 16, 18 and 45.

## Surgical treatment

Precursor lesions of the cervical cancer can be treated through local distructive methods or excision. The local distructive methods (cryotherapy, electrodiathermy, laser vaporization) are no more a standard therapy because they do not allow the histopathological examination of the specimen.

**Fig 1:**
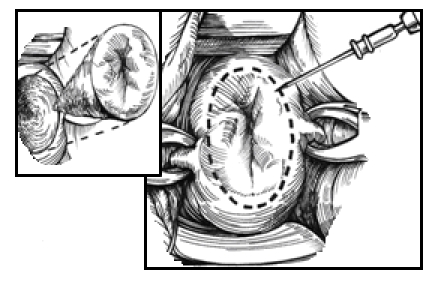
Needle excision of the transformation zone (NETZ).

Excisional methods are loop/needle electrosurgical excision procedures, laser excision and cold knife excision. Loop electrosurgical excision (LEEP) can be done as an outpatient procedure, with local anesthesia. Needle electrosurgical excision of the transformation zone (NETZ) has the advantage of excising the lesion as a single piece, easier to be histopathologically evaluated and allowing the optimal tailoring of the specimen to be resected (height and wide) [14]. The use of cold knife leaves the borders of the specimen clean, but the complications are more frequent. Postoperative complications are hemorrhage, infections and cervical stenosis, as well as the rise of the risk for premature delivery in a subsequent pregnancy (if the specimen height is over 1.5 cm). The frequency of these complications is very low [15, 16]. (Fig 1)

Cold knife conization has the advantage of preserving the specimen intact for the histopathological examination and allowing for a good evaluation of the tissue margins. Although the cure rate is the same as with the use of electrocautery, the frequency of complications is double.

Total histerectomy, abdominal or vaginal, represents a very rare indication in the therapy of precursor lesions of the cervical cancer. It is the choice when the lesion wasn't totally resected, persistent abnormal Pap smear, lesion extended to the vaginal vault, associated gynecologic pathology, difficult follow–up after conization.
